# Staphylococcus Aureus Bacteremia Due to Central Venous Catheter Infection: A Clinical Comparison of Infections Caused by Methicillin-Resistant and Methicillin-Susceptible Strains

**DOI:** 10.7759/cureus.16607

**Published:** 2021-07-24

**Authors:** Kazuhiro Ishikawa, Keiichi Furukawa

**Affiliations:** 1 Infectious Disease, St. Luke's International Hospital, Tokyo, JPN; 2 Infectious Disease, Asahi General Hospital, Asahi, JPN

**Keywords:** mssa, mrsa, central venous catheter infection, staphylococcus aureus, nosocomial infection

## Abstract

Objectives: *Staphylococcus aureus* bacteremia has a mortality rate of 20-40% and is mainly caused by central venous catheter (CVC) infection. We aimed to investigate differences between patients with methicillin-resistant *S. aureus *(MRSA) and methicillin-sensitive *S. aureus *(MSSA) bacteremia due to CVC infection.

Methods: This retrospective study, of data collected between August 2004 and March 2016 at a single Tokyo hospital, compared the clinical background characteristics, complications, and 60-day mortality rates with positive peripheral blood cultures and positive semiquantitative cultures. MRSA carrier is defined as those with a history of MRSA detection by skin, urine, or sputum culture.

Results: The median ages for the 17 MRSA and 19 MSSA patients were 72 and 55 years, respectively (P < 0.01). The occurrences of baseline disease (MRSA vs. MSSA) were 59% vs. 16% (P = 0.01), respectively, while those of complications, including septic shock, were 48% vs. 16% (P = 0.07), respectively. Catheter placement duration, time from fever onset to CVC removal, and time from fever onset to antimicrobial therapy initiation were similar in both groups. Sixty-day mortality rates were 35% and 5.3% (P = 0.04) in the MRSA and MSSA groups, respectively.

Conclusion: MRSA carriers and older patients were at higher risks of CVC infection than MSSA bacteremia patients. Patients with MRSA bacteremia had higher septic shock and 60-day mortality rates despite appropriate antimicrobial therapy.

## Introduction

*Staphylococcus aureus* bacteremia is mainly caused by central venous catheter (CVC) infection and is an important healthcare-associated infection [1,2]. Other risk factors include solid tumors, chronic kidney disease, history of hospitalization, and prolonged antimicrobial use. Approximately 20-50% of *S. aureus* isolated in Japanese medical facilities is methicillin-resistant *S. aureus* (MRSA) [3]. Generally, the associated mortality rate of *S. aureus *bacteremia is 20-40%, and many studies have reported that MRSA bacteremia has a higher associated mortality rate than that of methicillin-sensitive *S. aureus* (MSSA) bacteremia [2]. However, few studies have investigated the differences and prognoses of patients with MRSA and MSSA bacteremia secondary to CVC infection. We aimed to investigate the differences in clinical backgrounds, complications, and prognosis between MRSA and MSSA bacteremia secondary to CVC infection at St. Luke's International Hospital, Tokyo, Japan.

This article was previously published as a preprint in Research Square: Ishikawa K, Furukawa K, Hoshino E: Staphylococcus aureus bacteremia due to central venous catheter infection: a clinical comparison of infections caused by methicillin-resistant and methicillin-susceptible strains, January 11, 2021 (DOI: 10.21203/rs.3.rs-127180/v1).

## Materials and methods

This study was approved by the Institutional Review Board of St. Luke’s International Hospital in Tokyo, Japan (Number: 16-J011), and the need for informed consent was waived due to the retrospective nature of the study.

We retrospectively investigated the data of all patients who tested positive for MRSA or MSSA in peripheral blood cultures and semiquantitative cultures from CVC tip samples, taken within three days of the positive blood cultures between August 2004 and March 2016 at St. Luke’s International Hospital. Because our hospital did not perform quantitative blood culture and measure detection to time positivity, the CVC infection was only diagnosed using peripheral blood culture and semiquantitative culture. We included patients aged over 15 years with sufficient medical records and who were diagnosed with primary CVC infection. When 15 colonies or more were found, it was considered a positive semiquantitative culture. We excluded patients with secondary *S. aureus *bacteremia and end-stage malignancy.

The primary outcome was death from infection within 60 days of CVC removal. In this study, baseline characteristics included age (patients ≥ 65 years were defined as elderly), sex, MRSA carrier status (defined as those with a history of detection by skin, urine, or sputum culture), baseline diseases, ICU admission during hospitalization, surgery, total parenteral nutrition (TPN), steroid use, chemotherapy, and immunosuppressant use. Complications, including endophthalmitis, infective endocarditis, deep abscess, and septic shock were analyzed. Other factors, such as antimicrobial treatment, duration of CVC placement, the time lag from symptom onset (fever in most cases) to CVC removal, and time lag from symptom onset to effective antimicrobial therapy initiation (cloxacillin or cefazolin for MSSA and vancomycin {VCM} or daptomycin for MRSA) were also investigated. We also calculated the mortality rates within 60 days of removing the infected CVC and compared them between the MRSA and MSSA bacteremia patients.

The statistical analyses were divided into the MRSA and MSSA patient groups. In the univariate analysis, the Mann-Whitney U test was used for continuous variables while χ^2^ and Fisher's exact tests were used for categorical variables. The primary outcome was death within 60 days of CVC removal. Multivariate analysis was performed on MRSA, age, and sex.

As a sub-analysis, we investigated the minimum inhibitory concentration (MIC: μg/mL) of VCM in each MRSA strain isolated from patients with MRSA bacteremia who received VCM. We compared the VCM MIC values between patients who died and those who survived. We also investigated the relationship between VCM MIC values and the prognosis of MRSA bacteremia secondary to CVC infection.

## Results

**Figure 1:**
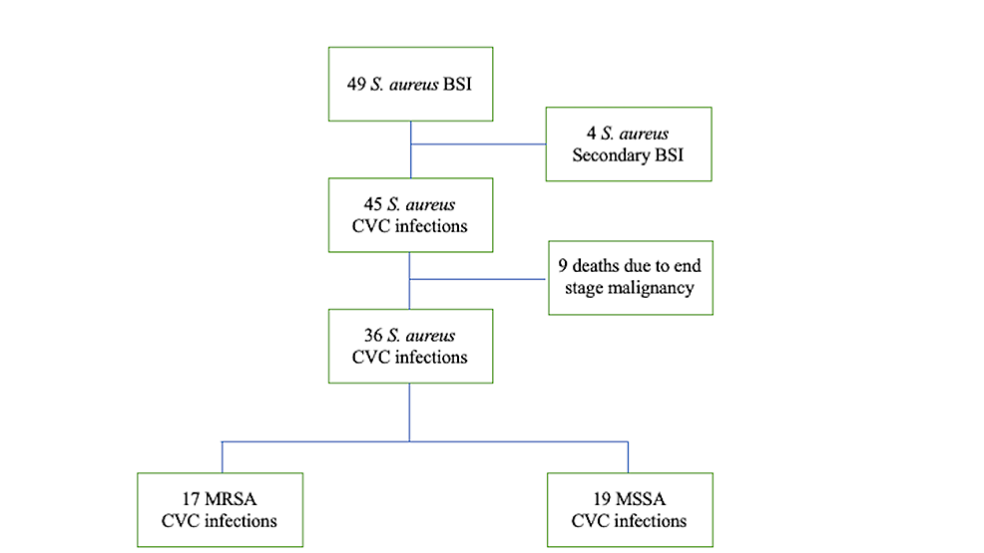
Process for identifying patients with Staphylococcus aureus bacteremia due to central venous catheter infection between August 2004 and March 2016 at St. Luke’s International Hospital, Tokyo, Japan

There were 49 patients with *S. aureus* bacteremia between August 2004 and March 2016 at St. Luke’s International Hospital. We excluded four patients with other primary infection sites and nine patients with end-stage malignancy. A total of 36 patients with *S. aureus* bacteremia due to CVC infection met the final inclusion criteria for the analysis. Of these, 17 patients (47%) had MRSA bacteremia and 19 (53%) had MSSA bacteremia (Figure 1).

Table 1 shows the baseline characteristics of the study population. The median age of all patients was 64 years (interquartile range: 45-83 years), and the median ages of patients with MRSA and MSSA were 72 ± 27 years and 55 ± 33 years (P < 0.01), respectively. Furthermore, 35% and 53% of patients with MRSA and MSSA bacteremia, respectively, were male (P = 0.30).

**Table 1:** Baseline characteristics of the patients with Staphylococcus aureus CVC infection between August 2004 and March 2016 at St. Luke’s International Hospital, Tokyo, Japan CVC: central venous catheter; MSSA: methicillin-susceptible *Staphylococcus aureus*; MRSA: methicillin-resistant *Staphylococcus aureus*; IQR: interquartile range

	*Staphylococcus aureus* (N=36)	MSSA (N=19)	MRSA (N=17)	P-value
Age: median (IQR) years Age (> 65), n (%)	64 (±19) 16 (44)	55 (±33) 6 (32)	72 (±27) 10 (59)	0.01 0.10
Male, n (%)	16 (44)	10 (53)	6 (35)	0.30
MRSA carrier, n (%)	13 (36)	3 (15.8)	10 (58.8)	0.01
Diabetes mellitus, n (%)	8 (22)	5 (26)	3 (18)	0.70
Malignancy, n (%)	7 (19)	5 (26)	2 (12)	0.41
Hematological malignancy, n (%)	6 (16.7)	2 (11)	4 (24)	0.39
Post-transplant, n (%)	2 (5.6)	0 (0)	2 (12)	0.22
Renal disease, n (%)	11 (31)	7 (37)	4 (24)	0.48
Liver disorder, n (%)	2 (5.6)	1 (5.3)	1 (5.9)	1.0
Cerebrovascular disease, n (%)	8 (22)	5 (26)	3 (18)	0.70
ICU stay, n (%)	12 (33)	6 (32)	6 (36)	1.0
Post-surgery, n (%)	8 (22)	4 (22)	4 (24)	1.0
Total parenteral nutrition, n (%)	17 (47)	7 (37)	10 (60)	0.32
Steroid user, n (%)	12 (33)	4 (22)	8 (48)	0.16
Chemotherapy, n (%)	4 (11)	3 (16)	1 (5.9)	0.61
Immunosuppressant, n (%)	4 (11)	1 (5.3)	3 (18)	0.33

At baseline, characteristics observed in the two groups were MRSA carrier status (10 {59%} MRSA vs. 3 {16%} MSSA, P = 0.01), diabetes mellitus (3 {18%} MRSA vs. 5 {26%} MSSA, P = 0.70), malignancy (2 {12%} MRSA vs. 5 {26%} MSSA, P = 0.41), renal diseases (4 {24%} MRSA vs. 7 {37%} MSSA, P = 0.48), steroid use (8 {48%} MRSA vs. 4 {22%} MSSA, P = 0.16), and TPN (10 {60%} MRSA vs. 7 {37%} MSSA, P = 0.32). No significant differences were observed in baseline diseases between the two groups.

Complications found in the two groups of patients included septic shock (8 {48%} MRSA vs. 3 {16%} MSSA, P = 0.07) and infective endocarditis (0 {0%} MRSA vs. 2 {11%} MSSA, P = 0.49) (Table 2). We found two cases of infective endocarditis in MSSA group which was diagnosed after CVC infection.

**Table 2:** Univariate analysis of complications and prognoses of MSSA versus MRSA bacteremia patients MSSA: methicillin-susceptible *Staphylococcus aureus*; MRSA: methicillin-resistant *Staphylococcus aureus*

	*Staphylococcus aureus* (N=36)	MSSA (N=19)	MRSA (N=17)	P-value
Endophthalmitis, n (%)	3 (8.3)	1 (5.3)	2 (12)	0.59
Infective endocarditis, n (%)	2 (5.6)	2 (11)	0 (0)	0.49
Deep abscess, n (%)	2 (5.6)	0 (0)	2 (12)	0.22
Septic shock, n (%)	11 (31)	3 (16)	8 (48)	0.07
60-day mortality rate, n (%)	7 (19)	1 (5.3)	6 (35)	0.04

Table 3 shows the management of CVC infection in the MSSA and MRSA groups. The duration of catheter placement in the two groups was 13.5 ± 8 days and 9.5 ± 20 days in patients with MRSA and MSSA bacteremia, respectively (P = 0.58). Time lags from fever onset to CVC removal in the two groups were < 1.0 day and 1.0 day in MRSA and MSSA patients, respectively (P = 0.71). The time lag from fever onset to effective antimicrobial therapy initiation was < 1.0 day in both groups (P = 0.7). All MRSA patients were treated with VCM. There were no differences in the duration of catheter placement, the time lag from fever onset to CVC removal, or time lag from fever onset to initiation of effective antimicrobial therapy between the two groups.

**Table 3:** Univariate analysis of the treatment of MSSA versus MRSA bacteremia patients MSSA: methicillin-sensitive *Staphylococcus aureus*; MRSA: methicillin-resistant *Staphylococcus aureus*; CVC: central venous catheter; IQR: interquartile range

	*Staphylococcus aureus* (N=36)	MSSA (N=19)	MRSA (N=17)	P-value
Duration of CVC placement, median (IQR) days	9.5 (20)	9.5 (20)	13.5 (8)	0.58
Time lag from symptom onset to CVC removal, median (IQR) days	1.0 (3)	1.0 (3)	0.0 (7)	0.71
Time lag from symptom onset to start of effective antibiotics, median (IQR) hours	0.0 (2)	0.0 (2)	0.0 (3)	0.07

Overall, 19% (7/36) of the patients died due to CVC-associated *S. aureus* bacteremia (MRSA or MSSA) such as sepsis, complications of circulatory failure such as heart failure, and respiratory failure such as nosocomial pneumonia within 60 days of CVC removal. From each group, 35% (6/17) and 5.3% (1/19) died due to MRSA and MSSA bacteremia, respectively. The 60-day mortality rate was significantly higher in patients with MRSA than MSSA bacteremia (35% vs. 5.3%, P = 0.04).

Table 4 shows the results of multivariate analysis of risk factors of 60-day mortality rate following CVC removal with adjustments for MRSA, males, and age > 65 years. There were no statistically significant differences between the two groups. However, the adjusted odds ratio (OR) was 8.2 (95% confidence interval {CI} 0.81-83; P = 0.08), which was lower than the crude OR (9.8 {95% CI 1.0-93; P = 0.05}) for the 60-day mortality of patients with MRSA bacteremia. In the sub-analysis, the VCM MIC results for MRSA isolated from all 17 patients were MIC < 1.0 μg/mL (65%, 11/17), MIC = 1.0 μg/mL (12%, 2/17), and MIC = 2.0 μg/mL (23%, 4/17). Altogether, 73% (8/11) of patients with MRSA bacteremia with VCM-susceptible strains (MIC < 1.0 µg/mL) survived, while 27% (3/11) died. In contrast, 50% (1/2) survival was observed among patients with MRSA bacteremia with moderate susceptibility to VCM (MIC =1.0 µg/mL). Lastly, 25% (1/4) survival was observed among patients with MRSA bacteremia and reduced VCM susceptibility (MIC = 2.0 µg/mL). However, these results were not statistically significant because of the small sample size (Table 5).

**Table 4:** Multivariate analysis of risk factors of 60-day mortality from CVC removal with adjustments for MRSA, age > 65 years, and males CVC: central venous catheter; MRSA: methicillin-resistant *Staphylococcus aureus*

	Crude OR (95% CI)	P-value	Adjusted OR (95% CI)	P-value
MRSA	9.8 (1.0-93)	0.05	8.2 (0.81-83)	0.08
Age (> 65)	1.9 (0.36-10)	0.46	1.4 (0.2-10)	0.71
Male	0.4 (0.07-2.6)	0.36	0.5 (0.06-3.8)	0.49

**Table 5:** VCM MIC value (μg/mL) in deceased and surviving MRSA bacteremia patients VCM: vancomycin; MIC: minimum inhibitory concentration; MRSA: methicillin-resistant *Staphylococcus aureus*

VCM MIC (μg/mL)	Patients who died (n=7)	Patients who survived (n=10)	Total (n=17) (% of total MRSA)	60-day survival rate (%)
2.0	3	1	4 (23%)	1/4 (25%)
1.0	1	1	2 (12%)	1/2 (50%)
< 1.0	3	8	11 (65%)	8/11 (73%)

## Discussion

The results of this study showed that patients with MRSA CVC infection were older than the patients with MSSA CVC infection. MRSA CVC infection was observed at a significantly higher rate in patients who were MRSA carriers compared to those who were not MRSA carriers. There was no significant difference in baseline diseases between patients with MRSA and MSSA bacteremia.

In both the MRSA and MSSA bacteremia groups, the infected CVCs were removed, and effective antimicrobial agents were started early; there was no significant difference in this amount of time. The patients with MRSA bacteremia showed a significantly higher 60-day mortality rate than those with MSSA bacteremia. Additionally, worse prognoses were found in MRSA bacteremia patients who were infected with a strain less susceptible to VCM (MIC 2.0 μg/mL), the MIC of which is the upper limit of VCM susceptibility, compared with MRSA bacteremia patients infected with a VCM-susceptible strain (MIC < 1.0 μg/mL) [4-8]. However, this result was not statistically significant due to the small sample size. Further studies with larger sample sizes are required to confirm any association.

According to the Infectious Diseases Society of America (IDSA) guidelines, daptomycin (6 mg/kg/day) or VCM is recommended as the first-line therapy for uncomplicated MRSA bacteremia without infective endocarditis [9]. However, the efficacy and bactericidal activity of VCM in treating MSSA bacteremia may be less than that of β-lactam antibiotics, such as cefazolin or cloxacillin [10].

In the multivariate analysis, the MRSA group showed no statistically significant difference in the 60-day mortality rate; however, the adjusted OR was lower than the crude OR. It was suggested that there might be other risk factors for death within 60 days in the MRSA group other than MRSA bacteremia alone. A previous study on *S. aureus* infections showed that comorbidities, such as septic shock, might be significant risk factors [2]; this should be investigated in future studies.

This study had several limitations. Firstly, we extracted data of patients from our database according to the definition of CVC infection by the catheter-related bloodstream infection (CRBSI) guidelines from IDSA [11] or the central line-associated bloodstream infection (CLABSI) guidelines from the Centers for Disease Control and Prevention [12]. However, at St. Luke’s International Hospital, the definition of CVC infection became available to clinicians after 2014, when the differential time to positivity and quantitative cultures were used. In this study, a patient was considered to have CVC infection when a central catheter, such as CVC, central venous port, or peripherally inserted central catheter, was placed in the central vein, and *S. aureus* was detected simultaneously in the blood culture and the catheter tip, using semiquantitative culture. Secondly, this was a single-center retrospective study; thus, there could have been selection bias. Lastly, there was a small sample size, therefore, the variables of multivariate analysis were limited. In the future, prospective observational research using CRBSI and CLABSI guidelines should be conducted at multiple facilities.

## Conclusions

Further studies are warranted to investigate whether patients with MRSA bacteremia due to CVC infection caused by strains with reduced susceptibility to VCM (MIC = 2.0 µg/mL) would have a worse prognosis compared with patients with MRSA bacteremia with VCM-susceptible strains (MIC < 1.0 µg/mL) under VCM therapy. Additionally, future investigations should focus on the possibility of improving prognosis using daptomycin at its therapeutic dose, which could have a more potent antibacterial activity, especially against MRSA infection with strains of reduced VCM susceptibility.

In conclusion, our study showed that patients with MRSA bacteremia secondary to CVC infection had a higher 60-day mortality rate than those of MSSA under antimicrobial therapy with VCM. Measures to prevent MRSA induced by CVC infection, and earlier, more effective antimicrobial therapy are challenges to address in future studies.
